# Contraction of Fingers

**Published:** 1880-08

**Authors:** 


### CONTRACTION OF FINGERS.

At a meeting of the Medical Society of London, Mr. William Adams exhibited a number of casts of contracted fingers. Many were due to contraction of the palmar fascia and its digital prolongations. Such were cured by multiple subcutaneous divisions of the fascia and immediate extension. Mr. Adams regarded it as usually commencing in chronic gouty inflammation and thickening of the fascia. These cases were compared with the cast of a case of finger contraction, following lacerated wound of the wrist, and depending on contraction of the tendons. He also showed a cast of a remarkable case in which the little finger was severely contracted by a web of skin extending from the point of the finger to its base. The President referred to the case of a gentleman aged eighty, who had suffered from contraction in both hands. Mr. Gant had remedied this in the right hand by tenotomy and extension. Mr. Spencer Watson asked for evidence of a gouty origin of the disease, and alluded to Mr. Hutchinson's observations of the concurrence of thickening of the sclerotic (and glaucoma) with Dupuytren's contraction. Dr. Fothergill had frequently noted these contractions in gouty subjects, and almost invariably found them symmetrical. Mr. Owen alluded to a case of this disease in a gentleman, aged thirty, in which the middle finger of the right hand was contracted, and where he had operated by dividing the fascial band, applying a splint and gradual extension. Dr. Gilbert Smith, referring to Mr. Adams' remark as to the rare occurrence of the disease among women, said he had lately seen a lady with both hands affected by these contractions. He also knew of an instance where three brothers, all of them gouty, suffered in this manner.

